# New Drugs from Marine Organisms in Alzheimer’s Disease

**DOI:** 10.3390/md14010005

**Published:** 2015-12-25

**Authors:** Patrizia Russo, Aliaksei Kisialiou, Palma Lamonaca, Rossana Moroni, Giulia Prinzi, Massimo Fini

**Affiliations:** 1 Clinical and Molecular Epidemiology Division, IRCCS “San RaffaelePisana” Via di Valcannuta, 247, RomeI-00166, Italy; aliaksei.kisialiou@sanraffaele.it (A.K.); palma.lamonaca@gmail.com (P.L.); rossana.moroni@sanraffaele.it (R.M.); giulia.prinzi@sanraffaele.it (G.P.); 2 Scientific Direction, IRCCS “San RaffaelePisana” Via di Valcannuta, 247, Rome I-00166, Italy; massimo.fini@sanraffaele.it

**Keywords:** marine drugs, Alzheimer’s disease, mechanisms of activity, clinical/preclinical studies, bryostatin, new drugs

## Abstract

Alzheimer’s disease (AD) is a multifactorial neurodegenerative disorder. Current approved drugs may only ameliorate symptoms in a restricted number of patients and for a restricted period of time. Currently, there is a translational research challenge into identifying the new effective drugs and their respective new therapeutic targets in AD and other neurodegenerative disorders. In this review, selected examples of marine-derived compounds in neurodegeneration, specifically in AD field are reported. The emphasis has been done on compounds and their possible relevant biological activities. The proposed drug development paradigm and current hypotheses should be accurately investigated in the future of AD therapy directions although taking into account successful examples of such approach represented by Cytarabine, Trabectedin, Eribulin and Ziconotide. We review a complexity of the translational research for such a development of new therapies for AD. Bryostatin is a prominent candidate for the therapy of AD and other types of dementia in humans.

## 1. Introduction

Right now, 46.8 million persons in the world are suffering from dementia and it is expected that this number will increase to 74.7 million in 2030 and 131.5 million in 2050 [1]. Alzheimer’s disease (AD) is the main cause of dementia in the elderly [2]. AD is a progressive, continuous and incurable brain disorder leading to increase severe disability such as memory loss (amnesia), minimal to no communication (aphasia), the inability to perform activitiesofdaily living (ADL) (apraxia), the impairment of the sensory input (development of agnosias**)**. In briefly, AD is a multifactorial neurodegenerative disorder that affects cognition (memory, thinking, and language abilities), quality of life and self-sufficiency in elderly [2]. AD is strictly related to aging, indeed the majority of cases (≥ 90%) are initially diagnosed among persons ≥ 65 years of age (AD with late onset—LOAD) [1]. Two percent to ten percent of cases diagnosed before the age of 65 years (AD with early onset—EOAD). Adominant EOAD caused by several genetic mutations [3]. In particular, genes involved in the production of the amyloid β (Aβ) peptides such as amyloid precursor protein (APP), Presenilin 1 (PSEN1), and 2 (PSEN2) may account for as much as 5%–10% of the EOAD incidence [3]. The allele ε4 of Apolipoprotein E (APOE) is considered an established genetic risk factor for both EOAD and LOAD [3,4]. APOEε4 homozygous carriers show eightfold elevation of risk of AD compared to the general population. However, up to 75% of APOEε4 heterogeneous subjects do not progress to AD during their lifetime, and on the other hand, up to 50% of AD patients aren’t APOEε4 carriers [3,4]. APOE is involved in lipid transport as well as in Aβ peptides transport [4]. At least 21 novel genetic risk *loci* has been emerged from recently performed genome-wide association studies (GWAS) of AD and massive parallel resequencing [3]. At the pathological level intracellular neurofibrillary tangles (NT: microtubule–associated protein tau (τ)) and extracellular amyloid plaques characterize AD brains [5]. Additionally, amyloid angiopathy, age-related brain atrophy, synaptic pathology, white matter rarefaction, granulovacuolar degeneration, and neuron loss are observed in AD post-mortem brain samples [5]. AD is an extensive neurodegenerative disease starting from the entorhinal cortex (limbic regions). Moreover, a post-mortem AD brain tissue autopsy reveals an impressive shrinkage in almost all neocortical areas, as well as a loss of subcortical structures such as the *substantianigra*, are largely spared. The molecular mechanisms that trigger sporadic LOAD, for the most part, are unknown. AD is a multifactorial disorder with a great number of leading mechanisms that support the postulation of different molecular etiological hypotheses [6,7,8,9,10,11,12,13,14,15,16,17,18].

The most well-supported scientific hypotheses are:

(i) “The amyloid cascade hypothesis”. For decades the hypothesis was the main “framework” for AD research. The pathological accumulation of Aβ, as amyloid plaques, frequently observed in AD brains [6], was considered the main etiopathology cause. The increased Aβ accumulation, according to this hypothesis, triggers a cascade of events leading to synaptic dysfunction, memory loss and structural brain damage in AD advanced stages. The hypothesis that Aβ peptides are the causal factors of AD is now considered an oversimplification. Consequently, a linear toxicity model (increased Aβ deposition which in turn increases a brain damage) is incorrect. However, the possible Aβ role is to trigger other downstream events, such as τ aggregation. The failure of Aβ-targeted clinical trials in AD patients supports the hypothesis that Aβ peptides may be recurrent contributors in the AD process, but it is neither necessary, nor sufficient [6];
(ii) “The cholinergic hypothesis”. The hypothesis is based on the observation ofsignificant loss in cholinergic signaling such as a severe loss of brain white matter with the reduction of cholinergic neurons of the basal forebrain (*i.e.*, Acetylcholine (ACh), nicotine/muscarinic binding sites (nicotinic/muscarinic receptor: nAChR, mAChR)) observed in post-mortem cerebral cortex of AD patients [7]. A significant reduction of the number of nicotine and ACh binding sites in cerebral cortex of AD patients supports a decrease in the number of both nAChR and mAChR. Moreover, the activity of choline acetyltransferase (ChAT) and acetylcholinesterase (AChE) is decreased. The two enzymes are involved in ACh synthesis/degradation: ChAT transfers an acetyl group from the coenzyme (acetyl-CoA) to choline yielding ACh while AChE catalyzes ACh breakdown. Consequently, any failure in the cholinergic system is strictly linked to attention, learning and memory deficit;
(iii) “The glutamatergic hypothesis”. The hypothesis is based on the gradual deterioration of proper synaptic functioning through GluN2A-containing *N*-methyl-d-aspartate receptors (NMDARs) and the development of excitotoxicity through GluN2B-containing NMDARs. Alteration in NMDARs activity may involve Aβ-induced synaptic impairment, spine loss and neurodegeneration [8];
(iv) “The mitochondrial hypothesis” [9]. The hypothesis predicts that mitochondrial dysfunctions trigger energy metabolism impairment, with excessive reactive oxygen species (ROS) formation and consequent DNA damage [10];
(v) “The metabolic hypothesis” is based on the assumption that mitochondrial dysregulation up-regulates the oxidative phosphorylation (OXPHOS) activity (known as “inverse Warburg effect”) [11];
(vi) “The τ hypothesis”. The hypothesis is based on the observation that τ dysfunction (abnormal levels, hyperphosphorylation, or ubiquination), in the absence of amyloid pathology, is sufficient to cause synaptic and neuronal loss [12];
(vii) “The memory kinase hypothesis” is based on the involvement of Protein Kinase C (PKC) in acquisition and modification of dendritic spines, in neurite retraction and in synaptic plasticity [13] (For details see Bryostatin-1 section);
(viii) “The neuro-inflammation hypothesis”. The hypothesis implies an innate immune response characterized by the release of inflammatory mediators [14,15];
(ix) “The clearance systems hypothesis” is based on Aβ clearance failure. In briefly, an excess deposition of Aβ peptides results from an imbalance between their production and clearance; in both EOAD and LOAD, as well as at the prodromal stage [16];
(x) “The Cognitive Reserve (CR) hypothesis”. The hypothesis is proposed to explain the gap between the brain insult and the pathological manifestations. The CR includes two elements: brain (*i.e.*, brain size, synaptic count, and dendritic branching) and cognitive (*i.e.*, neural and compensation reserve) reserve. Two components of the reserve work together and protect the brain from AD [17].
(xi) “The disconnection hypothesis”. The hypothesis is based on the disrupted functional connectivity in AD brains association area [18,19,20].

Probably, the above mechanisms may work altogether through interactions between genetic, molecular and cellular events [21]. For example, the α7-nAChR may be a convergent point for several hypotheses. Aβ binds to α7-nAChR with a high affinity. Aβ induces τ phosphorylation through α7-nAChR activation. Aβ concentrations, Aβ aggregation conditions, as well as possible presence of β2 nicotinic subtype in the composition of the heteromer α7β2-nAChR, may cause activation or inhibition of α7-nAChR with consequent neuroprotection or neurotoxicity effect [22,23]. α7-nAChR activation of macrophage, microglia and neuron induces the JAK2/STAT3 anti-inflammation pathway [23]. Moreover, soluble oligomeric Aβ peptides engage α7-nAChR in astrocytes, in which glutamate release, in turn, activates neuronal NMDARs and consequent synaptic damage [24].

There are only four currently FDA/EMA-approved drugs. Two drugs namely: donepezil, rivastigmine (ATC codes N06DA02, N06DA03, respectively) are based on the cholinergic and/or glutamatergic hypotheses. These drugs (reversible acetylcholinesterase inhibitors (AChEI)) act through inhibition of AChE and butyrylcholinesterase (BChE). Galantamine (ATC code N06DA04) is a competitive, reversible α7-nAChR allosteric inhibitor [22]. Memantine (ATC code N06DX02) is NMDARs antagonist [25]. AChEI inhibit AChE by breaking down ACh, thus, increasing ACh effects, which, hypothetically, elevate cholinergic signaling in neurons and limit inflammation [15,25].

No drugs has been proved to be effective in the treatment of Mild Cognitive Impairment (MCI, a prodromal state of AD). Consequently, AChEI and memantine were not approved by FDA/EMA for MCI subjects [26]. None of these drugs change or block disease progression; they may only ameliorate symptoms in a restricted number of patients and for a restricted period of time [25,27]. Tacrine (ATC code N06DA01) is a reversible AChEI, currently discontinued because of liver toxicity, was the first drug receiving FDA approval in 1993 [28], donepezil—in 1996. Consequently, memantinereceived FDA approval in 2003, galantamine—in 2004 and, finally, rivastigmine—in 2006. In the last 22 years only five AD drugs were FDA-approved, in comparison to 29 new anticancer agents approvedin 2013–2015 [29]. It is obvious that there is a pressing need to new AD/MCI drugs. Thus, in 2015 WHO includes among health research priorities: “*Implement and take the necessary steps towards the ambition to identify a cure or a disease-modifying therapy for dementia by 2025 as adopted by the G8 Summit in December 2013*” [30].

## 2. Drugs from Marine Organisms

Marine organisms live in different underwater habitats (environment) characterized by specific chemical and physical properties such as water salt concentrations, pressure, temperature (including extreme), light penetration, oxygen concentrations and radiation exposure, and ocean currents. The distinctive marine environment dictates the marine organism’s adaptation involving their structural (or morphological), physiological and behavioral adaptations [31,32]. Marine organisms are comprised in six different kingdoms: Bacteria, Protozoans, Chromists (including Seaweeds), Fungi, Plants, although few types flourish in the marine environment, and Animals including jellyfish, sponges, sea spiders, bryozoans, mussels, sea stars, fish and whales. Marine organisms or marine organisms with associated microbial communities can synthesize extremely structural different metabolites used to immobilize and capture prey and to defend against predators. These compounds range from small peptides (*i.e.*, conopeptides of 7–27 amino acids in length) and enzymes to more complex secondary metabolites (*i.e.*, ecteinascidin, a tetrahydroisoquinoline alkaloid), which show significant bioactivities that efficiently disturb vital physiological systems, in particular, those linked to movement, respiration and circulation. These molecules enclose the potential to become a lead in AD innovative drug discovery [32,33,34,35,36,37,38,39]. In this field of research a breakthrough discovery is represented by the FDA/EMA-approved drugs: Cytarabine (from *Cryptotethyacrypta*), Trabectedin (from *Ecteinascidia turbinate*) and Eribulinmesylate (from *Halichodriaokadai*) discovered and developedas anticancer agents, and Ziconotide (from *Conus magus*) approved for treatment of neuropathic pain [33,34,35,36,37,38,39].

In this review, selected examples were reported in order to exemplify the development of marine-derived compounds for neurodegenerative diseases, specifically, for AD. The emphasis was done on new compounds with their relevant biological activities. Accordingly, new compounds without biological activity or, at least, not tested in cells were therefore not included.

### 2.1. Bryostatin-1

**Figure 1:**
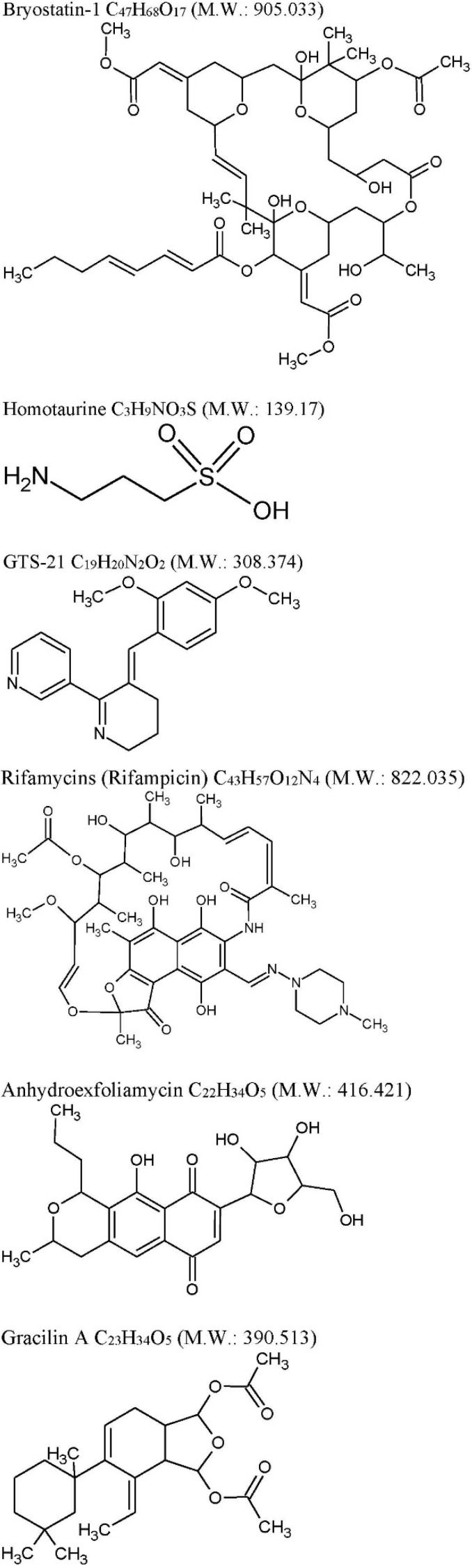

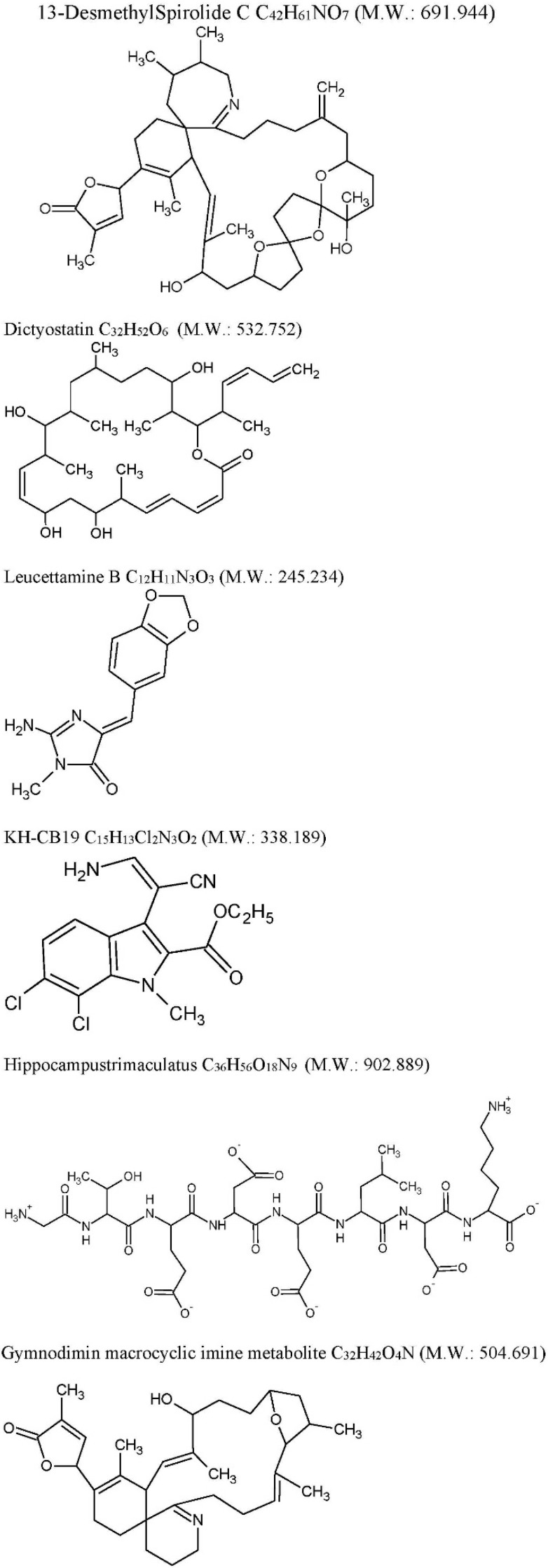
Chemical structures. Chemical structures were drawn using ACD/ChemSketch^®^.

The pharmacology of Bryostatin-1 (Bry-1, C_47_H_68_O_17_ M.W. = 905.04, Figure 1) is emblematic [40,41] of marine drugs potential, since Bry-1 is exploited in non-correlated different diseases such as cancer, HIV and neurodegenerative diseases. Bry-1 was initially isolated from the extract of *Bugulaneritina* (or brown bryozoans, natively distributed in tropical and subtropical waters, now widespread globally through vessels hulls attachment to the ships) at the end of the 60s by George Pettit [42]. Bry-1 is a macrolide lactone characterized by 11 chiral centers. Currently, Bry-1 is obtained in total synthesis [43,44]. Cancer is the largest area of pharmacological exploitation of Bry-1 and its derivatives including apoptotic restoration, multidrug-resistance circumvention, immune system stimulation, and drugs synergism. Although as a single agent Bry-1’s activity was disappointing. Promising results were obtained in phase II clinical trials when Bry-1 was administered in combinations with cytotoxic agents such as, for example, Bry-1 and Cisplatin for the treatment of metastatic or unresectable stomach cancer [45].

Among the new rising important pharmacological activities of Bry-1 there is the ability to reactivate latent viral infection [46], also in human astrocytes, through the PKC/NF-κB-dependent mechanism [47]. The goal of the ongoing interventional randomized double blind dose-finding trial is to evaluate two different doses of Bry-1 on HIV-1 latency and reservoir in HIV-1 infected patients receiving antiretroviral treatment [48].

Bry-1 is a potent modulator of PKC [49]. PKC comprises eight isoforms (conventional: α, βI/βII, γ; and novel: δ, ε, η, θ) enclosing the regulatory C1 domains [49]. The rings of Bry-1 molecule, after binding to C1 domain, protrude forming a cap. Bry-1, although is a hydrophilic molecule, which binds strongly to PKC, with a potency similar to that of phorbol ester (a canonical hydrophobic ligand), causing PKC-α, β and δ down-regulation and no PKC-ε and RasGRP3 (RAS guanyl releasing protein 3 (calcium and DAG-regulated)) induction [50,51]. In brief, Bry-1 awakens a fast short activation and self-phosphorylation of PKCs that consecutively induces PKCs membrane translocation with succeeding PKCs down-regulation. The down-regulation of PKC-δ isozyme shows a distinctive biphasic pattern: at low concentrations—a down-regulation and at higher concentrations—a mechanism of protection [51]. This property contributes in making Bry-1 an attractive drug for pharmaceutical development.

Preclinical studies show that Bry-1 is able to:

(i) enhance spatial learning and long-term memory in rats, mice, rabbits and the nudibranch (*Hermissenda*) [52,53];
(ii) increase spinophilin (regulatory subunit of protein phosphatase-1 catalytic subunit highly enriched in dendritic spines) and synaptophysin (major synaptic vesicle protein p38), synaptic proteins levels causing synapses structural changes [52];
(iii) exert neuroprotective effects on AD transgenic mice [54];
(iv) improve memory (measured as reduction in latency to escape, after oral Bry-1) in APP/PS1 (mice containing human transgenes for both amyloid precursor protein (APP), bearing the Swedish KM670/671NL (rs63751263, rs63750445) mutation and PSEN1 containing an L166P mutation (rs63750265), both under the control of the Thy1 promoter) transgenic mouse [55];
(v) reduce Aβ levels in monomeric Aβ-treated cells “*in vitro*” [56];
(vi) reduce Aβ levels in Tg2576 AD mouse (mice overexpressing a mutant form of APP (isoform 695)) and aged rat recovery [57];
(vii) recover neurotrophic activity and synapses loss [57];
(viii) prevent neuronal apoptosis [57];
(ix) inhibit τ phosphorylation by GSK-3β inhibition [57];
(x) enhance synaptogenesis, leading cognitive deficits recovery [57].

Currently, three human trials are ongoing with the aim to exploit the role of Bry-1 in AD (Table 1) [58,59,60]. Neurotrope, Inc. (OTCQB: NTRP), the company producing Bry-1, announces positive top-line results from a randomized, double-blind, placebo-controlled, single dose Phase IIa clinical trial (ClinicalTrials.gov identifier NCT02221947) evaluating Bry-1 for the treatment of AD showing preliminary safety and tolerability data and no serious adverse events [61]. Moreover, the company (NTRP) has been granted orphan drug designation by the FDA for Bry-1 in the treatment of Fragile X Syndrome (FXS) [62]. FXS is the principle cause of “inherited intellectual disability” including moderate to severe learning disabilities, behavioral disorders, and cognitive impairment, and of autism or autism spectrum disorders. FXS is caused by a partial or a full mutation of the FMR1 gene [63]. Currently, there are no FDA-approved drugs for FXS. Bry-1 is also in preclinical studies in Niemann-Pick type C (NPC) mice with the aim to confirm previous “*in vitro*” studies suggesting efficacy in correcting NPC cholesterol transport defect [64]. NPC is a rare (the highest incidence 1% in Nova Scotia) devastating genetic disorder in children characterized by progressive neurodegeneration [65]. Although NPC is a really rare autosomal recessive disease, it shows some neuropathological similarities with AD, such as neurofibrillary tangles and deregulated Aβ metabolism. According to Malnar *et al.*, the strongest common denominator is the link to genes involved in cholesterol metabolism [66]. Additional studies on similarities and differences between AD and NPC may support the use of Bry-1 in both diseases. However, overall Bryostatin studies sustain the concept that the paradigm “*one-disease-one-drug-one-target*” is now “*history*”.

**Table 1:** Marine organisms drug derivatives in human clinical trials in Alzheimer’s Disease (AD).

ClinicalTrials.Gov Identifier	Title of the Trial	Study Design/Endpoint Classification	Primary Purpose	Ref
Bryostatin-1: C_47_H_68_O_17_ M.W. 905.04 from *Bugulaneritina* (or brown bryozoans).				
NCT00606164 Verified: January 2008 by Blanchette Rockefeller Neurosciences Institute.	Safety, Efficacy, Pharmacokinetics, and Pharmacodynamics Study of Bryostatin-1 in Patients With AD.	Randomized Interventional Safety/Efficacy Study Double Blind *	Find out single-dose safety. This study is also being done: (1) to determine how effective a single dose of Bry-1 is in the treatment of AD; (2) to find out what happens to Bry-1 once it enters the body by measuring the levels of Bry-1 in blood; (3) to measure PKC-C in the blood.	[58]
NCT02221947 Terminated Verified: April 2015 (not specified)	Study to Evaluate the Preliminary Safety, Efficacy, PK and PD of Bryostatin-1 in Patients With AD.	Randomized Safety/Efficacy Study Double Blind*	Evaluate the safety and tolerability following a single intravenous dose	[59]
NCT02431468 Verified: April 2015 by Neurotrope Bioscience, Inc.	A Study Assessing Bryostatin-1 in the Treatment of Moderately Severe to Severe AD.	Randomized Safety/Efficacy Study Double Blind*	To compare different doses for the treatment of moderately severe to severe AD. The study is 28 weeks in duration, including a safety and efficacy 30 days evaluation after the last dose of the study drug.	[60]
Homotaurine: (Tramiprosate) C_3_H_9_NO_3_S M.W. 139.17 from a red alga *Grateloupia livid*				
NCT00314912 Last verified: July 2007 Bellus Health Inc.	Open-Label Extension of the Phase III Study With Tramiprosate (3APS) in Patients With Mild to Moderate AD.	Randomized, double-blind, placebo-controlled, parallel-group study conducted at 67 study centers across the United States and Canada	Evaluate the long-term safety. Secondary Outcome Measures: To provide additional long-term data on the efficacy of Tramiprosate (3APS). No significant treatment effect	[67,68]
--	Homotaurine induces measurable changes of short latency afferent inhibition in a group of MCI individuals.	10 MCI patients at 100 mg for 4 weeks	SLAI cortical inhibitory circuit changes, no SICI changes, unable to induce changes of the LTP/LTD mechanisms	[70]
GTS-21: C_19_H_20_N_2_O_2_ M.W. 308.374, anabaseine synthetic derivative from *Nemertines* (ribbon worms).				
NCT00414622 Last Updated: April 18, 2007	A Double Blind, Placebo-Controlled Randomized Study to Compare the Safety and Tolerability of GTS-21 (25 mg TID, 50 mg TID, 75 mg TID and 150 mg TID) When Administered Daily for 28 Days to Participants With Probable AD.	Randomized Double-Blind	Endpoint Classification: Safety/Efficacy Study Primary Purpose: Treatment The study amperes as completed, however no results are present	[72,73]
Rifamycins: C_43_H_57_O_12_N_4_ M.W. 822.036 previously known to be produced only by soil actinobacteria *Amycolatopsis* is produced by marine bacteria—*Salinispora* isolated from the marine sponge *Pseudoceratinaclavata*.				
--	A multicenter, blinded, randomized, factorial controlled trial of doxycycline and rifampin for treatment of AD: the DARAD trial.	DARAD study: multicenter, blinded, randomized, placebo-controlled factorial doxycycline and rifampin	Neither rifampin nor doxycycline provided any benefit to patients with AD.	[74]
--	A randomized, controlled trial of doxycycline and rifampin for patients with AD.	Randomized, triple-blind, controlled trial.	Possible therapeutic role in patients with mild to moderate AD	[75]

* Subject, Caregiver, Investigator, Outcomes Assessor; LTD: prolonged long-term depression; LTP: long-term potentiation (synaptic plasticity); SICI: intracortical inhibition; SLAI: short latency afferent inhibition, a neurophysiological measure of central cholinergic transmission.

### 2.2. Drugs in Ongoing Clinical Trials

At the moment, several marine natural products and their derivatives (Figure 1) are under evaluation as novel drugs for the treatment of neurological disorders, including AD (see Table 1).

Homotaurine (tramiprosate, a small aminosulfonate compound, Figure 1) obtained by red marine algae was evaluated in phase III clinical trials in mild-to-moderate AD [67], showing no enough clinical efficacy [68]. However, looking at secondary endpoints of the study such as lower decline in memory function and reduction in global cognitive decline in APOEε4 allele carriers subgroups of patients, some disease-modifying effects are reported [69]. Homotaurine in preclinical models shows neuroprotective effect inhibiting Aβ activity and by γ-aminobutyric acid type A receptor affinity [69]. A small study was conducted on 10 MCI patients with the aim to study the effects of homotaurine on motor cortical excitability (Table 1) [70]. Homotaurine induced changes of short latency afferent inhibition (SLAI) SLAI measures the impairment of central cholinergic functions “*in vivo*” and consists “in the inhibition of the Motor Evoked Potentials (MEPs) by afferent sensory impulses” [71]. It has been suggested that Homotaurine-dependent effects, related to changes of cortical GABA transmission, may ameliorate the cholinergic transmission [70].

Homotaurine protects neurons both “*in vitro*”(NGF-differentiated PC12 cells and primary cortical neurons) and “*in vivo*” (in rats subjected to the intraluminal filament model of MCAO: Middle cerebral artery occlusion) against ischemic stroke, through disruption of the interaction between PSD95 and nNOS and inhibition of nNOS translocation [76]. The scaffolding protein postsynaptic density-95 (PSD95) binds to both NMDARs and nNOS at excitatory synapses.

Homotaurine was evaluated in a single blind, randomized, controlled study (24 patients *versus* 13 controls) to evaluate safety and efficacy in Parkinson’s disease (PD) patients with cognitive impairment. After six months of treatment no difference was reported between groups and no adverse effect. A beneficial effect of Homotaurine was observed only on excessive sleepiness in patients with PD [77].

GTS-21 (Figure 1) a synthetic derivative of anabaseine is in phase II for participants with probable diagnosis of AD [72] but with discouraging results. GTS-21 is a partial agonist of α4β2- and α7-nAChR subtypes, able to significantly activate α7 [73].

Rifampicins (Figure 1), a class of broad-spectrum antibiotics, previously known to be produced only by soil actinobacteria *Amycolatopsis* is also produced by the marine bacteria *Salinispora* isolated from the marine sponge *Pseudoceratinaclavata* [78]. It has been proposed that Rifampicin may exert a neuroprotective effects through both scavenging free radicals mechanisms and inhibition of Aβfibrillar formation (reviewed in [79]). The decrease of intracellular accumulation of Aβ_1–40_ seams associated with P_gp_ up-regulation Rifampicin-mediated (reviewed in [79]). On the basis of these preclinical evidences Rifampicin, in association with doxycycline, was evaluated on AD patients (Table 1), however, with discouraging results [74,75].

### 2.3. Drugs in Preclinical Evaluations

Actually, FDA/EMA-approved drugs work on cholinergic (donepezil, rivastigmine, galantamine) or glutamatergic (memantine) hypothesis. Randomized clinical trials based on the amyloid cascade hypothesis completely failed. Consequently, it was a leitmotiv for a search for new promising targets and marine compounds discussed in all the Review. Among marine derivative drugs, currently under study, we selected only those compounds able to interfere to that molecular processes possible involved in causatives of AD. Specifically, we describe drugs showing activity in *(i)* τ inhibition such as Anhydroexfoliamycin, Gracilins, 13-desmethyl spirolide-C and Dictyostatin; *(ii)* CDC2-like kinase inhibition, such as Leucettamine B, and KH-CB19; *(iii)* Aβ aggregation inhibition, such as peptides HTP-1 and Gymnodimin. Table 2 and Figure 1 show drugs currently in preclinical development in AD mouse-model system or in cell cultures [80,81,82,83,84,85,86]. Compounds, such as AChEI assayed only in “*in vitro*” enzymatic assay, although, potentially interesting, are not discussed.

**Table 2:** Marine organisms drug derivatives in pre-clinical trials in AD.

Drug	Source	Target	Cellular/Animal Model	Effect	Ref
τ inhibition					
Anhydroexfoliamycin: C_22_H_24_O_8_ M.W. 416.421	*Streptomyces exfoliatus* from marine soil	GSK3β mediated by the JNK pathway	3xTg-AD mice	GSK3β inhibition τ, phosphorylation reduction	[78]
Gracilins: C_23_H_34_O_5_ M.W. 390.513	*Spongionella* sp.	Mitochondrial targeting through the induction of Nrf2 translocation. BACE1 and ERK inhibition, τhyperphosphorylation reduction.	3xTg-AD mice	After chronic intraperitoneal treatments, a preliminary behavioral test pointed a positive trend on learning and spatial memory of mice treated with these compounds. Moreover, *in vivo* assays confirmed the previous results. Amyloid-β42 and hyperphosphorylated tau levels were decreased after treatments and the ERK inhibition was also observed.	[79]
13-desmethyl spirolide-C (SPX): C_42_H_61_NO_7_ M.W. 691.944 Spirolides*	*Alexandriumostenfeldii/peruvianum*dinoflagellates	Decrease GSK-3β and ERK.	3xTg mice cortical neurons	Glutamate-induced neurotoxicity inhibition both in control and 3xTg neurons.	[74]
Dictyostatin: C_32_H_52_O_6_ M.W. 532.751	*Spongia* sp. and Caribbean sponge family *Corallistidae*	MT-stabilizing agent	CD1 mice	MT-stabilization in the brain one week after 5 mg/kg i.p. administration	[75]
CDC2-like kinase inhibitors					
Leucettamine B: C_12_H_11_N_3_O_3_ M.W. 245.234	*Leucettamicroraphis* Haeckel (Calcarea) sponge	CLK1, Dyrk1A and Dyrk2 inhibition and CLK3 moderate inhibition.	Human U937 cell membrane	--	[80]
KH-CB19: C_15_H_13_Cl_2_N_3_O_2_ M.W. 338.188 dichloroindolylenaminonitrile derived from bauerine C	*Dichothrixbaueriana* blue-green alga	CLK1 and Dyrk1A potent inhibitor.	Inhibition of human recombinant CLK1 (148 to 484 amino acids) expressed in *Escherichia coli* BL21.	--	[80]
Amyloid-β Aggregation Inhibitors					
Trimaculatus-derived neuroprotective peptides HTP-1: Gly-Thr-Glu-Asp-Glu-Leu-Asp-Lys: C_36_H_56_O_18_N_9_ M.W. 902.889	*Hippocampus trimaculatus* (seahorse)	--	PC12	Aβ42-induced neuronal death protection. Bcl-2 up-regulation.	[81]
Gymnodiminmacrocyclic imine metabolite: C_32_H_45_O_4_N M.W. 504.691	*Kareniaselliformis* (formerly named *Gymnodiniumselliformis*) (dinoflagellate)	Antagonize human α7-nAChR expressed in Xenopus oocytes	3xTg mice cortical neurons	Aβ intracellular accumulation, τhyperphosphorylation reduction, Glutamate-induced neuronal death prevention	[82]

* Not affect the steady-state levels of neither the M1 and M2 muscarinic nor the α7-nAChR, while it decreased the amplitude of ACh-evoked responses and increased ACh levels in 3xTg neurons.

## 3. Concluding Remarks

The success stories of Cytarabine (ATC code L01BC01), Trabectedin (ATC code L01CX01), Eribulin (ATC code L01XX41) and Ziconotide (ATC code N02BG08) [33,34,35,36,37,38,39] as well as the rich pipeline of clinical, preclinical and tool compounds from marine organisms, especially from microorganisms, (see Table 1 and Table 2) make clear that the marine world offers a great reservoir of potential investigational drugs. The specificity of singular marine habitats yields compounds structurally unique. On the other hand, the use of these compounds is limited by their chemical complexity and natural scarcity amounts. The chemical synthesis is the only approach to obtain unlimited quantities, however, the high complexity of certain structures may make synthesis difficult, and, in some cases, impractical. Indeed, in 1990 Corey won the Nobel Prize in Chemistry “*for his development of the theory and methodology of organic synthesis*” that then allows the synthesis of Ecteinascidin (Trabectedin) [87] originally purified by the tunicate *Ecteinascidia turbinate* only in very small amounts.

In spite of the observation that a great number of compounds with different interesting pharmacological profile arrive to preclinical studies, the majority of them did not succeed the clinical studies. Indeed, Cummings *et al.* [88] examining the Clinicaltrials.gov database, a public website that records ongoing clinical trials, for the period time 2002–2012 retrieve 413 AD human trials: 124 Phase I, 206 Phase II, and 83 Phase III. In this decade only three agents reached the FDA/EMA approval. The study of Cummings *et al.* [88] definitively proved that the development of an effective drug for AD is very difficult and the pipeline is the lowest in comparison with any other therapeutic area [29].

The likelihood to create different effective bioactive products starting by a scaffold obtained by a marine natural product is now possible through technology improvements both in harvesting samples, and in purifying, and characterizing products. Therefore, the ocean resources may be regularly exploited for designing and producing of drug discovery pipeline.
